# Infant Parasympathetic and Sympathetic Activity during Baseline, Stress and Recovery: Interactions with Prenatal Adversity Predict Physical Aggression in Toddlerhood

**DOI:** 10.1007/s10802-017-0337-y

**Published:** 2017-08-07

**Authors:** J. Suurland, K. B. van der Heijden, S. C. J. Huijbregts, S. H. M. van Goozen, H. Swaab

**Affiliations:** 1 Department of Clinical Child and Adolescent Studies, Leiden University, Wassenaarseweg 52, Room 4A03, Box 9555, 2300 RB Leiden, The Netherlands; 2 Leiden Institute for Brain and Cognition, Leiden University, Leiden, The Netherlands; 3 School of Psychology, Cardiff University, Cardiff, UK

**Keywords:** Aggression, Stress reactivity, Respiratory sinus arrhythmia, Pre-ejection period, Prenatal risk, Infancy

## Abstract

Exposure to prenatal adversity is associated with aggression later in life. Individual differences in autonomic nervous system (ANS) functioning, specifically nonreciprocal activation of the parasympathetic (PNS) and sympathetic (SNS) nervous systems, increase susceptibility to aggression, especially in the context of adversity. Previous work examining interactions between early adversity and ANS functioning in infancy is scarce and has not examined interaction between PNS and SNS. This study examined whether the PNS and SNS moderate the relation between cumulative prenatal risk and early physical aggression in 124 children (57% male). Cumulative risk (e.g., maternal psychiatric disorder, substance (ab)use, and social adversity) was assessed during pregnancy. Parasympathetic respiratory sinus arrhythmia (RSA) and sympathetic pre-ejection period (PEP) at baseline, in response to and during recovery from emotional challenge were measured at 6 months. Physical aggression and non-physical aggression/oppositional behavior were measured at 30 months. The results showed that cumulative prenatal risk predicted elevated physical aggression and non-physical aggression/oppositional behavior in toddlerhood; however, the effects on physical aggression were moderated by PNS and SNS functioning. Specifically, the effects of cumulative risk on physical aggression were particularly evident in children characterized by low baseline PNS activity and/or by nonreciprocal activity of the PNS and SNS, characterized by decreased activity (i.e., coinhibition) or increased activity (i.e., coactivation) of both systems at baseline and/or in response to emotional challenge. These findings extend our understanding of the interaction between perinatal risk and infant ANS functioning on developmental outcome.

**Electronic supplementary material:**

The online version of this article (doi:10.1007/s10802-017-0337-y) contains supplementary material, which is available to authorized users.

## ᅟ

The earliest expressions of aggression are already apparent in infancy (Hay et al. 2010; Tremblay et al. 2004). Although physical aggression is known to peak at age 2 and 3, and then to decline over the preschool period (Alink et al. 2006), there is evidence that relatively high levels of aggressive behavior during early development predict persistent and severe aggressive and antisocial behavior over the course of childhood (NICHD Early Child Care Research Network 2004), and a range of other problems including low academic achievement and poor social relationships (Campbell, Spieker, Burchinal, Poe, and The NICHD Early Child Care Research Network 2006). Research has highlighted that adversity experienced during prenatal development can have long-lasting effects on children’s development (Monk et al. 2012; Rice et al. 2010). Childhood aggressive behavior has been linked to a number of different risk factors during the prenatal period such as high levels of stress, anxiety and depression or antisocial behavior (Hay et al. 2011; O'Connor et al. 2002; Rice et al. 2010), smoking (Huijbregts et al. 2008), low socioeconomic status, low educational attainment, and early entry into parenthood (NICHD Early Child Care Research Network 2004; Tremblay et al. 2004). Although exposure to maternal risk factors during prenatal development is highly correlated with continued exposure during postnatal development (Monk et al. 2012), there is evidence that prenatal stress predicts childhood antisocial behavior irrespective of postnatal circumstances (Rice et al. 2010). Notably, previous work has shown a dose-dependent relation between the presence of multiple risk factors and child adjustment (Appleyard et al. 2005).

It is generally acknowledged that children differ in their physiological susceptibility to these early adversities (Boyce and Ellis 2005). A growing number of studies in children and adolescents have examined interactions between adversity and measures of autonomic nervous system (ANS) functioning in predicting the development of aggression (El-Sheikh and Erath 2011). The ANS plays an important role in emotion regulation (Porges 2007), and abnormal ANS functioning has been linked to aggression and externalizing behavior (Van Goozen et al. 2007). During infancy, the ANS is rapidly developing which is associated with increased responsiveness to environmental influences (Porges and Furman 2011). Yet, we know little about how the ANS interacts with early adversity in infancy. In the present study, we examined whether measures of ANS functioning in infancy moderated the relation between cumulative prenatal risk and early physical aggression.

### The Autonomic Nervous System and Aggression

Maturation of the ANS during infancy provides the foundation for emotional and behavioral regulation observed later in development (Porges and Furman 2011). The ANS is comprised of the sympathetic (SNS) and parasympathetic (PNS) nervous system. The SNS initiates the “fight/flight” response by increasing heart rate and respiration. In contrast, the PNS has an inhibitory effect on the SNS and its role is to maintain homeostasis and to regulate recovery following stress by decreasing heart rate and respiration. PNS activity is often assessed by respiratory sinus arrhythmia (RSA), the heart rate variability at the frequency of respiration (Cacioppo et al. 1994), which is thought to index the neural control of the heart via the vagus nerve (Porges 2007). In response to stress, RSA levels are assumed to decline, indicating withdrawal of the ‘brake’ on the SNS allowing for flexible responding to stress, active engagement with the environment, and coping with mild to moderate stressors (see Porges and Furman 2011 for a review). If withdrawal of the PNS is not sufficient to manage a stressor, SNS activity is expected to increase in order to prepare the body for more active stress responses.

The majority of research examining stress reactivity in young children has focused on RSA or global measures of autonomic functioning like heart rate without specific assessments of the SNS. SNS functioning can be measured by the pre-ejection period (PEP), which represents the time between the onset of the heartbeat and ejection of blood into the aorta (Cacioppo et al. 1994). Although assessment of SNS activity by skin conductance level (SCL) is more common, PEP is considered to be a more pure and direct indicator of cardiac SNS activity and can be reliably measured in infants (Alkon et al. 2011; Quigley and Stifter 2006).

Reduced parasympathetic control, as indicated by low baseline RSA and low RSA reactivity to stress, and attenuated SNS activity (measured by SCL or PEP) at baseline and in response to stress and reward, have been associated with externalizing problems in children and adolescents (Beauchaine et al. 2007; El-Sheikh and Erath 2011; Graziano and Derefinko 2013). However, these associations may be different in clinical samples as increased RSA reactivity has been reported in children with clinical externalizing problems (Beauchaine et al. 2007). Further, the link between RSA and externalizing behavior is less clear in infants and toddlers, and higher baseline RSA has been linked to more negative reactivity (Fox et al. 2000). Furthermore, there is some evidence that relations between RSA and externalizing problems do not emerge until after the preschool age (Beauchaine et al. 2007).

Several theoretical frameworks posit that the effects of ANS functioning on developmental outcome occur not directly, but in interaction with environmental factors (Boyce and Ellis 2005; El-Sheikh and Erath 2011). Indeed, empirical evidence shows that low baseline RSA and low RSA reactivity exacerbate the relation between environmental risk (e.g., marital conflict, parental drinking problems, domestic violence) and children’s externalizing behavior (El-Sheikh 2001, 2005a; El-Sheikh et al. 2001). Studies investigating interactions between adversity and SNS activity indicate that either very low or very high baseline levels of SCL and high SCL reactivity may increase the risk of aggression and externalizing behavior in the context of adversity (El-Sheikh 2005b; El-Sheikh et al. 2007).

It is clear that ANS functioning has important implications for the association between adversity and the development of aggression. However, few studies to date have investigated this issue in infancy and the findings have been inconsistent. Two recent studies suggest that higher (rather than lower) baseline RSA and RSA reactivity predict the development of problem behavior in infants exposed to a more negative caregiving environment (Conradt et al. 2016; Conradt et al. 2013). One other study examined interactions between chronic maternal depression, overcrowded housing and infant RSA and PEP reactivity in predicting externalizing problems at age 7 (Waters et al. 2016). The results showed that low RSA reactivity in combination with chronic maternal depression was related to more externalizing problems, whereas high PEP reactivity was associated with lower levels of externalizing problems in the context of chronic maternal depression. However, a study in toddlers found no evidence of an interaction between environmental quality and RSA reactivity in the prediction of aggressive behavior (Eisenberg et al. 2012).

### Interaction between Stress Systems

Adaptation to stressful contexts requires a delicate balance in the operation of both the PNS and SNS (Porges 2007), and the synergistic action of both systems determines the effectiveness of regulation (Berntson et al. 1991). Reciprocal autonomic activation, in which the PNS and SNS are oppositely activated, with increased activation of one system and decreased activation of the other, reflects a coordinated response in which both systems either increase or decrease physiological arousal to support responses to environmental demands. However, nonreciprocal activation of the PNS and SNS, with increased or decreased activation of both systems at the same time, is possible (Berntson et al. 1991).

Reciprocal ANS activation, particularly reciprocal SNS activation (i.e., increased SNS activation and decreased PNS activation) in response to stress, is presumed to be normative (Alkon et al. 2011; Salomon et al. 2000), and linked better emotion regulation in young children (Stifter et al. 2011). Conversely, nonreciprocal activation of PNS and SNS may indicate a breakdown in stress regulation, in which either the PNS or SNS fails to perform its adaptive function in response to stress (Porges 2007). Indeed, El-Sheikh et al. (2009) have shown that children with decreased PNS and SNS activation (i.e., *coinhibition*) or increased PNS and SNS activation (i.e., *coactivation*) exhibited higher levels of externalizing problems in the context of marital conflict, compared to children showing reciprocal activation of the two systems (i.e., *reciprocal PNS activation* and *reciprocal SNS activation*). Similar findings were reported in the context of maltreatment predicting aggression among girls (Gordis et al. 2010).

Until now, there have been no studies that have examined the interaction between the PNS and SNS in infancy as potential moderator of the effects of early adversity on developmental outcome in toddlerhood. Because there may be differences in autonomic influence across development from infancy to childhood (Beauchaine et al. 2007), there is a need to further understand how the interaction between the PNS and SNS in infancy may increase or decrease susceptibility to early adversity.

### The Present Study

In the present study we examined the interactive effects of prenatal adversity and infant ANS regulation as longitudinal predictors of physical aggression in toddlerhood. The study adds to the existing literature in several ways: 1) We measured both PNS and SNS functioning and their interaction. Previous studies in infants have primarily examined baseline RSA as a moderator of early adversity on developmental outcome. However, there is much inconsistency in the literature regarding the relation between ANS functioning and aggressive behavior. Examining measures of both PNS and SNS functioning, as well as their interactions could improve our understanding of the role of the ANS in the development of aggression. As far as we know, only one previous study examined PNS and SNS reactivity in infants, but this study did not test their interactive effects (Waters et al. 2016). 2) We also investigated whether the expected interactions between early adversity and both PNS and SNS functioning were specific for physical aggression as opposed to non-physical aggression/oppositional behavior. Physical aggression and oppositional behavior are both part of the externalizing spectrum representing correlated constructs of behavior problems. However, as there is evidence that physical aggression and non-physical/oppositional behavior are associated with different developmental processes (Burt 2012), and alterations in ANS functioning are linked specifically to aggressive but not to non-aggressive/oppositional behavior (Baker et al. 2013), it is important to consider the possibility of differential physiological susceptibility between these two constructs. 3) We were specifically interested in cumulative prenatal risk since cumulative risk models are considered to be more powerful than single risk models in predicting problem behavior (Appleyard et al. 2005). 4) We measured parasympathetic RSA and sympathetic PEP at baseline, in response to and during recovery from stress. Baseline (or resting) measures of RSA and PEP are thought to reflect neural integrity and readiness to respond to environmental stressors (Beauchaine 2001). However, reactivity and recovery measures may be stronger predictors of later behavioral outcomes (Fox et al. 2000). Notably, measures indexing autonomic recovery from stress have been underrepresented in the current literature (El-Sheikh and Erath 2011). 5) We investigated interactions between RSA and PEP within dimensions (i.e., RSA baseline x PEP baseline etc.) and across dimensions (e.g., RSA baseline x PEP response, and RSA response x PEP recovery) as baseline and reactivity measures of RSA and PEP can combine in different ways to buffer or exacerbate effects of early adversity (El-Sheikh et al. 2009; Gordis et al. 2010). This approach allows us to examine a diverse set of PNS x SNS interactions that may moderate the effects of adversity on physical aggression and non-physical aggression/oppositional behavior later in development.

We hypothesized that the interaction between PNS and SNS functioning would moderate the association between cumulative prenatal risk and physical aggression, such that nonreciprocal activation of the PNS and SNS (i.e., increased or decreased activation of both systems), would exacerbate the relation between cumulative prenatal risk and physical aggression, whereas reciprocal activation of the PNS and SNS (i.e., increased activation of one system and decreased activation of the other), would attenuate the relation between cumulative prenatal risk and physical aggression. Further, we expected that these moderating effects would be specific for physical aggression as opposed to non-physical aggression/ oppositional behavior. Finally, in the analyses we controlled for the effects of temperament and behavioral distress and demographic and obstetric characteristics.

## Method and Materials

### Participants

Data were collected as part of the Mother-Infant Neurodevelopment Study (MINDS) – Leiden, which is an ongoing longitudinal study of Dutch mothers and their first-born children focusing on neurobiological and neurocognitive predictors of early behavior problems. We oversampled families based on the presence of one or more risk factors (see criteria under Cumulative risk) to obtain sufficient variance in children’s early behavioral problems. Detailed information about the study and sample selection has been reported elsewhere (Smaling et al. 2015; Suurland et al. 2017). The study was approved by the ethics committee of the Department of Education and Child Studies at the Faculty of Social and Behavioral Sciences, Leiden University, and by the Medical Research Ethics Committee at Leiden University Medical Centre. Informed consent was obtained from all individual participants included in the study.

A priori power analysis (Faul et al. 2009) indicated that a sample size of approximately 100 would provide sufficient power (*ρ* = 0.80, *α* = 0.05) to test the proposed regression models and to find an effect size comparable to previous studies (El-Sheikh et al. 2009; Gordis et al. 2010). A total of 136 mothers were originally enrolled in the study at T1 (third trimester of pregnancy). Twelve mothers dropped out between T1 and T2 (6 months post-partum), and another 23 mothers dropped out between T2 and T3 (30 months post-partum), resulting in a sample of 101. The main reasons for families dropping out were inability to be contacted, moving away or too busy. Sample attrition was unrelated to demographic variables or any dependent measures (*ps* > 0.05). However, mothers who dropped out were more often single, *χ* ^2^(1) = 8.41, *p* < 0.05. To increase our sample size and power, we used multiple imputation for participants who had data on the home-visits at T1 and T2, but did not complete the laboratory visit at T3 (see Data analysis for more details). This resulted in a sample of 124, with (*ρ* = 0.90, *α* = 0.05).

The mean age of the children was 6.02 months (*SD* = 0.41, range 5–7 months) at T2 and 30.05 months (*SD* = 1.00, range 28–33 months) at T3. At T1, mothers were on average 22.91 years (*SD* = 2.12, range 17–27 years), approximately 93.5% had a partner (84.7% was married or living with a partner), and 29.8% had a high educational level (Bachelor’s or Master’s degree). Families were predominantly Caucasian (85.5%).

### Procedures

During the prenatal home-visit (between 26 and 40 weeks gestation, *M* = 29.78, *SD* = 3.63), mothers were screened for the presence of risk factors based on an interview and multiple questionnaires (Smaling et al. 2015). The protocol during the six-month home-visit, included attachment of cardiac monitoring equipment to the infant’s chest and back. Baseline ANS functioning while at rest was measured during a two-minute relaxing movie while the infant was lying on a blanket, followed by two procedures designed to elicit physiological responses to social stress (Still Face Paradigm) and frustration (Car seat). The social stress and frustration tasks were administered with a break in between to limit carry over effects. Infants were only assessed in the next procedure when they were calm and displayed no distress. The home-visits were scheduled at a time of the day when mothers deemed their infant to be most alert.

The Still Face Paradigm (SFP; Mesman et al. 2009) is a well-established social stress paradigm comprising a sequence of three 2-min episodes during which the mother is asked to interact normally with the infant (SFP baseline), then withhold interaction (SFP social stress), and then resume interaction (SFP recovery; for a more detailed description of the SFP, see Suurland et al. 2017). The Car Seat (CS) task, adapted from the Laboratory Temperament Assessment Battery Pre-locomotor version (Lab-TAB;Goldsmith and Rothbart 1999), was used to measure infant ANS and behavioral response to a frustrating event. Following a 2-min baseline (CS baseline), the mothers placed their infants in a car seat with straps firmly attached and stood 1 m away from their child. After 1 min of restraint (CS frustration), a 2-min recovery period (CS recovery) followed in which mothers were allowed to hold their child and interact as they normally would. Mothers were instructed to remain neutral and refrain from comforting or speaking to the child during the CS frustration episode.

During the 30-month laboratory visit, several tasks were performed and mothers completed multiple questionnaires. For the purpose of the current study, only maternal reports of physical aggression and non-physical aggression/oppositional behavior were examined.

### Measures

#### Cumulative Risk (T1)

Cumulative prenatal risk consisted of 10 criteria that were scored as present (1) or absent (0); current psychiatric disorder(s) with the Dutch version of the Mini- International Neuropsychiatric Interview (MINI-plus; Van Vliet et al. 2000), substance use (alcohol, tobacco and/or drugs) during pregnancy, no secondary education, unemployment, self-reported financial problems, limited or instable social support network, single status, and maternal age < 20 years (see for a more elaborate description of these criteria Smaling et al. 2015). The cumulative risk score was computed as the sum of risk factors present (maximum number of risk factors was 10), with *M* = .73, *SD* = 1.05 (range 0–5). There were 71 mothers with no risk factors, 28 with one risk factor, 15 with two risk factors, 8 with three risk factors, and two with respectively four and five risk factors. Because there were only two participants with respectively four and five risk factors, the presence of three, four or five risk factors was collapsed into one group with ≥3 risk factors. The prevalence of the different risk factors among participants with one or more risk factors (42.7%) was: 52.8% current psychiatric diagnosis, 5.7% alcohol, 41.5% smoking, 1.9% drugs, 15.1% single status, 11.3% unemployed, 5.7% no secondary education, 13.2% financial problems, 9.4% limited social support, 15.1% age < 20 years.

#### ANS Parameters (T2)

Parasympathetic RSA and sympathetic PEP were monitored with the Vrije Universiteit Ambulatory Monitoring System (VU-AMS 5 fs; De Geus et al. 1995; Willemsen et al. 1996). The VU-AMS device continuously recorded electrocardiogram (ECG), and impedance cardiogram (ICG) measures; basal thorax impedance (Z_0_), changes in impedance (dZ), and the first derivative of pulsatile changes in transthoracic impedance (dZ/dt). The ECG and dZ/dt signal were sampled at 1000 Hz, and the Z_0_ signal was sampled at 10 Hz. The VUDAMS software suite version 2.0 was used to extract mean values RSA and PEP across baseline (2 min), SFP baseline (2 min), SFP social stress (2 min), and SFP recovery (2 min), and CS baseline (2 min), CS frustration (1 min), and CS recovery (2 min).

R-peaks in the ECG, scored by the software, were visually checked and adjusted when necessary. RSA was derived by the peak-trough method (De Geus et al. 1995; Grossman et al. 1990), which combined the respiration, obtained from filtered (0.1–0.4 Hz) thoracic impedance signal, and inter beat interval (IBI) time series to calculate the shortest IBI during heart rate acceleration in the inspiration phase and the longest IBI during deceleration in the expiration phase (De Geus et al. 1995). RSA was defined as the difference between the longest IBI’s during expiration and shortest IBI’s during inspiration. Automatic scoring of RSA was checked by visual inspection of the respiratory signal from the entire recording. Because RSA was skewed at baseline, the emotional challenge tasks, and recovery, its natural logarithm (lnRSA) was used in the analyses.

PEP is the time interval between the onset of the ventricular depolarization (Q-wave onset) and the onset of left ventricular ejection of blood into the aorta (B-point on the Dz/dt complex; De Geus et al. 1995). Average dZ/dt waveforms were derived by the software. PEP was automatically scored from the Q-wave onset on the ECG and the B-point on the dZ/dt waveform. Each automated scoring was checked and corrected manually when necessary (Riese et al. 2003). Wave forms which were morphologically distorted and could not be visually corrected, were discarded. The procedure of interactive visual scoring was done independently by two trained raters; inter-rater reliability (intraclass correlation ICC) was 0.95.

LnRSA and PEP response and recovery scores on the SFP and CS were computed as standardized residualized change scores which represent the standardized residuals from the linear regressions of response and recovery scores on the preceding score to provide a simple change score adjusted for their initial value (El-Sheikh et al. 2009). The standardized residualized change scores for lnRSA and PEP during response and recovery on the SFP were significantly correlated with the standardized residualized change scores for lnRSA and PEP during response and recovery on the CS (*rs =* 0.24 to 0.28, with *ps* = 0.021 to 0.009). Therefore, the residualized change scores were averaged to create four indices: lnRSA response and PEP response (average SFP and CS) and lnRSA recovery and PEP recovery (average SFP and CS). Negative values reflect lnRSA and PEP decreases (i.e., greater PNS suppression and greater SNS activation respectively), while positive values reflect lnRSA and PEP increases (i.e., greater PNS activation and greater SNS suppression respectively).

#### Behavioral Distress (T2)

Infant behavioral distress (i.e., intensity of whining, fussing or crying) was coded by four trained raters from videotaped recordings according to scales of the Mother Infant Coding System (Miller et al. 2002) for all SFP episodes, and the Lab-TAB (Goldsmith and Rothbart 1999) for the CS frustration episode. Shifts in distress across the SFP episodes were in the expected direction (i.e., more distress during the social stress and recovery episodes compared to the baseline episode; Mesman et al. 2009), and there were significant correlations between distress and PNS and SNS activity (i.e., more distress during the social stress episode correlated with stronger PNS suppression from baseline to social stress, and more distress during the recovery episode correlated with stronger SNS activity from social stress to recovery; see Suurland et al. 2017). The scores for distress on the SFP and CS correlated significantly (*r* = 0.240, *p* < 0.05) and a composite score was created based on the standardized average of both scales. A subset of recordings (15% of the sample) was double-coded to assess inter-rater reliability. Intraclass correlation (ICC) was 0.999 on the SFP social stress episode and 0.950 on the CS frustration episode.

#### Temperament – Distress to Limitations (T2)

The short form of the Revised Infant Behavior Questionnaire (IBQ-R; Gartstein and Rothbart 2003) assesses 14 domains of temperament and was completed by the mother. We used the ‘Distress to limitations’ subscale (7 items) as a measure of fussing, crying or showing distress. The items were scored on a 7-point scale from *never* (1) to *always* (7). Internal consistency (Cronbach’s alpha) in the present sample was 0.74.

#### Physical Aggression (T3)

Mothers reported on their child’s physical aggression using the 11-item Physical Aggression Scale for Early Childhood (PASEC;(Alink et al. 2006). The PASEC items were originally derived from Tremblay et al. (1999) and the physical aggression items of the Child Behavior Checklist (CBCL) 1 ½ -5 yr. (Achenbach and Rescorla 2000). Mothers scored whether their child has shown certain physically aggressive behaviors (e.g., ‘hits’, ‘kicks’, ‘destroying things’) during the past two months on a 3-point Likert scale (0 = *not true* to 2 = *very true or often true*). A total score for physical aggression was calculated by summing item scores (range 0–22). The PASEC showed sufficient reliability in a sample of 2253 children recruited at 12, 24 and 36 months (Alink et al. 2006). The reported mean scores for the 24-month cohort were 3.20 (*SD* = 3.06), and 2.99 (*SD* = 3.07) for the 36-month cohort. Internal consistency (Cronbach’s alpha) in the present sample was. 73.

#### Non-physical Aggression/ Oppositional Behavior (T3)

The CBCL 1 ½-5 yr. (Achenbach and Rescorla 2000) was used to assess non-physical aggression/oppositional behavior. Mothers indicated whether their child displayed any of the 100 behavioral descriptions in the last two months on a 3- point Likert scale (0 = ‘not true’ to 2 = ‘very true or often true’), with higher scores indicating higher levels of problem behavior. We used the DSM-oriented Oppositional Defiant disorder subscale, consisting of six items (range 0–12) measuring oppositional and hard-to-manage behavior (e.g., ‘stubborn’, ‘temper tantrums’, ‘uncooperative’). The reliability and validity of the CBCL have been confirmed in several studies (e.g., Koot et al. 1997). Internal consistency (Cronbach’s alpha) for the Oppositional Defiant problems subscale in this sample was 0.77.

### Data Analysis

All variables were examined for outliers and violations of specific assumptions applying to the statistical tests used. Variables with values that exceeded >3*SD* from the group mean were recoded to the next extreme value within 3*SD* from the mean (0.7% of the ANS data across all SFP and CS episodes).

Hierarchical regression analyses were conducted to examine the interactive effects among cumulative risk, lnRSA (baseline, response or recovery) and PEP (baseline, response or recovery) on physical aggression and non-physical aggression/oppositional behavior. In separate regression models the following interaction effects between lnRSA and PEP were examined: 1) lnRSA baseline x PEP baseline, 2) lnRSA response x PEP baseline, 3) lnRSA baseline x PEP response, 4) lnRSA response x PEP response, 5) lnRSA recovery x PEP response, 6) lnRSA response x PEP recovery, and 7) lnRSA recovery x PEP recovery. All variables were centered to their mean prior to analyses (Aiken and West 1991). Step 1 included cumulative risk, Step 2 included lnRSA and PEP, Step 3 included all two-way interactions between cumulative risk, lnRSA and PEP, and Step 4 included the three-way interaction between cumulative risk, lnRSA, and PEP. We reported and interpreted the main and interaction effects of cumulative risk and ANS variables from the full interaction model. Significant interaction effects were examined following procedures recommended by Aiken and West (Aiken and West 1991) by plotting regression lines at 0 risk factors and 1.6 risk factors (i.e., mean number of risk factors for the group of infants with ≥1 risk factors) and 1 SD above and below the mean for the moderators (lnRSA baseline/lnRSA response/lnRSA recovery, and PEP baseline/ PEP response/PEP recovery).

The scores on the cumulative risk variable were skewed to the right with 57% of the participants having no risk factors and 43% having one or more risk factors. Although the regression residuals did not show any skewing, we checked the consistency of our findings by conducting all regression analyses with the cumulative risk variable dichotomized at 0 versus 1 or more risk factors. The results these analyses did not change the pattern of findings (data not shown). We also tested whether the main and interactive effects were moderated by sex. Because this was not the case, we did not report these findings.

A total of 19 participants were missing ANS data at baseline or one or more episodes of the SFP and/or CS, and 23 were missing data on physical aggression and non-physical aggression/oppositional behavior at T3. Missing data were not systematically related to demographic and obstetric characteristics (ethnicity, sex, gestational age; *p*s > 0.250) or any of the main study variables (*p*s > 0.250). However, infants with more missing ANS data had higher birth weight (*r* = 0.20, *p* < 0.05). Missing ANS data at T2 and physical aggression and non-physical aggression/oppositional behavior data at T3 were imputed through multiple imputation resulting in an analytic sample of 124. The iterative imputation approach allows full use of the data and protects against biased estimates (Schafer and Graham 2002). A total of 10 imputed datasets were generated using MICE (Van Buuren 2012) in R, version 3.3.2. Although MI tends to yield conservative estimates, this approach with 10 imputations is 95% efficient with 50% missing information. Relative efficiency estimates exceeded 95% of each parameter. Informed by pooled estimates and variances across imputed datasets, analyses were completed using SPSS 21.0. All regression models were also estimated using only the complete cases (87 participants had complete data at T1, T2 and T3) and the main and interaction effects remained unchanged. Coefficients and standard errors are reported for the imputed data and the simple slopes were obtained from the complete case data.

## Results

### Descriptive Analyses

Descriptive statistics for lnRSA and PEP baseline, response and recovery variables are presented in Online Resource 1. LnRSA and PEP response and recovery levels on the SFP and CS were significantly different from zero: *t*(114) = 4.67, *p* < 0.001 for lnRSA SFP response, *t*(105) = 2.79, *p* < 0.01 for lnRSA CS recovery, *t*(99) = 2.53, *p* < 0.05 for PEP SFP response, *t*(96) = 3.18, *p* < 0.01 for PEP CS response, and *t*(91) = −2.48, *p* < 0.05 for PEP CS recovery), except for lnRSA CS response (*t*(108) = −0.07, *p* = 0.944), lnRSA SFP recovery (*t*(114) = −1.42, *p* = 0.158), and PEP SFP recovery *t*(94) = 0.05, *p* = 0.961.

Averaged across the SFP and CS challenge episodes, 63.6% of the sample showed a decrease in lnRSA (i.e., PNS suppression) and 60.7% exhibited a decrease in PEP (i.e., SNS activation) from baseline. Averaged across the SFP and CS recovery episodes, 46.2% of the sample showed an increase in lnRSA (i.e., PNS activation) and 52.7% showed an increase in PEP (i.e., SNS suppression) from the challenge episode. Thus, there was sufficient variability in infant lnRSA and PEP response to and recovery from challenge.

### Preliminary Analyses

Means, SDs, and correlations for the potential covariates and main study variables are presented in Table 1. For interpretation purposes, lnRSA and PEP raw change scores are used for means and SDs in Table 1; however, as noted, residualized change scores are used in the correlation and regression analyses. Cumulative risk was significantly associated with physical aggression (*r* = 0.38, *p* < 0.001), and non-physical aggression/oppositional behavior (*r* = 0.34, *p* < 0.01). Cumulative risk, physical aggression and non-physical aggression/oppositional behavior were not significantly related to baseline, response and recovery measures of lnRSA and PEP.

**Table 1:** Means, standard deviations and correlations among study variables

Variable	1.	2.	3.	4.	5.	6.	7.	8.	9.	10.	11.	12.	13.	14.	15.
1. Cumulative risk	-														
2. Ethnicity^a^	0.20*	-													
3. Infant sex^b^	0.04	0.09	-												
4. Gestational age	0.00	−0.02	0.05	-											
5. Birth weight (kg)	−0.17†	−0.06	−0.14	0.64***	-										
6. Behavioral distress	−0.11	0.04	−0.03	0.14	0.09	-									
7. Distress (IBQ-R) ^c^	0.08	0.04	−0.02	0.00	0.06	0.11	-								
8. lnRSA baseline	−0.00	−0.01	0.01	−0.08	−0.09	0.09	−0.06	-							
9. lnRSA response	−0.01	0.14	−0.05	0.02	0.07	−0.24**	−0.07	0.30**	-						
10. lnRSA recovery	−0.07	0.10	0.07	−0.18†	−0.16†	0.12*	−0.05	0.39***	−0.19*	-					
11. PEP baseline	−0.03	−0.08	−0.01	0.02	0.07	−0.04	−0.11	0.08	0.24*	−0.06	-				
12. PEP response	0.12	−0.10	0.18†	−0.07	−0.10	−0.06	−0.02	−0.24*	−0.04	0.03	−0.01	-			
13. PEP recovery	−0.06	0.14	0.00	0.13	0.14	0.08	−0.05	0.09	0.20*	−0.12	0.41***	−0.29**	-		
14. Physical aggression	0.38***	0.10	−0.14	0.09	−0.10	−0.05	0.05	−0.06	0.02	−0.03	−0.06	0.01	−0.06	-	
15. Non-physical aggression/ oppositional behavior	0.34**	0.09	−0.10	−0.04	−0.08	0.04	0.06	0.04	0.10	0.14	−0.11	−0.08	−0.07	0.45***	-
*N*	124	124	124	124	124	123	121	118	118	119	113	112	112	101	101
*M*	0.71	89.1%	57.4%	39.2	3.4	0.01	2.84	3.39	0.08	0.03	64.23	1.26	−0.61	2.50	4.88
*SD*	0.97			1.95	0.53	0.80	0.96	0.44	0.35	0.30	6.11	3.24	3.67	2.18	2.10

*lnRSA* natural logarithm of respiratory sinus arrhythmia, *PEP* pre-ejection period, ^a^ % Caucasian, ^b^ % male, ^c^ ‘Distress to limitations’ subscale of the Revised Infant Behavior Questionnaire (IBQ-R) - short form. Spearman correlations were used to compute correlations with cumulative risk and Pearson correlations were used for correlations between all other variables

† < 0.10, *p < 0.05, **p < 0.01, ***p < 0.001

Behavioral distress was significantly related to lnRSA response and recovery (respectively *r* = −0.24, *p* < 0.01, and *r* = 0.12, *p* < 0.05). Further, there were marginally significant correlations between birth weight and RSA recovery (*r* = −0.16, *p* = 0.087), and gestational age and RSA recovery (*r* = −0.18, *p* = 0.051. In preliminary analyses, we tested whether inclusion of these covariates changed the results from main regression analyses. Because this was not the case, we reported the analyses without the covariates.

### Regression Analyses

#### Physical Aggression

**Fig. 1:**
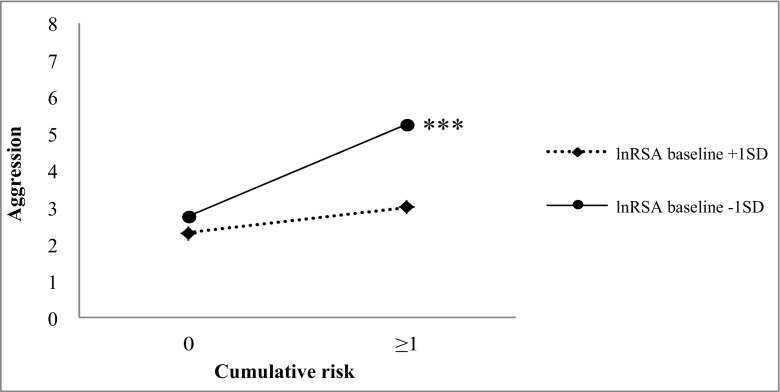
Two-way interaction between lnRSA baseline and cumulative risk, predicting physical aggression. Cumulative risk is plotted at 0 risk factors and 1.6 risk factors (this is the average number of risk factors present in infants with one or more risk factors), ***p < 0.001

In the hierarchical regression analyses predicting physical aggression (see Table 2), significant main effects, controlling for the effects of the other predictors included in step 1–4, were present for cumulative risk (*prs* = 0.41–0.51, *ps* < 0.001). Higher cumulative risk predicted higher levels of physical aggression. There were no significant main effects for lnRSA or PEP baseline, response or recovery. In model one, a significant two-way interaction effect was revealed between cumulative risk x lnRSA baseline (*pr* = −0.27, *p* < 0.05; although this interaction effect was significant in model one, it was marginally significant in model 2: *pr* = 0.23, *p* < 0.05) and cumulative risk x PEP baseline (*pr* = −0.23, *p* < 0.05; this two-way interaction effect was not further interpreted as it was moderated by lnRSA response in model three, see three-way interactions below). Examination of simple slopes (see Fig. 1) revealed that for infants with lower baseline lnRSA (−1 SD), higher cumulative risk predicted higher levels of physical aggression (*β* = 0.66, *p* < 0.001). Cumulative risk was not associated with physical aggression for infants with higher baseline lnRSA (+1 SD; *β* = 0.19, *p* = 0.152). None of the other two-way interaction effects between cumulative risk, lnRSA and PEP on physical aggression were significant.

**Table 2:** Interactions between lnRSA and PEP baseline and response moderate the association between cumulative risk and physical aggression

		Physical aggression		Non-physical aggression/oppositional behavior	
Step	Predictor	*b (se)*	*t*	*b (se)*	*t*
Model 1: LnRSA baseline x PEP baseline					
1	Cumulative risk	0.1.06 (0.23)	4.69***	0.63 (0.23)	2.76**
2	lnRSA	−0.52 (0.52)	−1.00	−0.01 (0.46)	−0.2
	PEP	0.00 (0.03)	0.04	−0.02 (0.04)	−0.52
3	lnRSA x PEP	−0.04 (0.04)	−0.81	−0.03 (0.05)	−0.59
	Cumulative risk x lnRSA	−1.31 (0.59)	−2.22*	−0.56 (0.52)	−1.07
	Cumulative risk x PEP	0.07 (0.03)	2.07*	0.01 (0.03)	0.39
4	Cumulative risk x lnRSA x PEP	0.08 (0.08)	0.95	0.05 (0.09)	0.61
Model 2: LnRSA baseline x PEP response					
1	Cumulative risk	0.99 (0.24)	4.07***	0.79 (0.26)	3.05**
2	lnRSA	−0.59 (0.51)	−1.17	0.10 (0.51)	0.20
	PEP	0.05 (0.30)	0.16	0.07 (0.27)	0.25
3	lnRSA x PEP	−0.21 (0.45)	−0.47	0.24 (0.50)	0.47
	Cumulative risk x lnRSA	−1.15 (0.61)	−1.87†	−0.17 (0.59)	−0.29
	Cumulative risk x PEP	0.02 (0.34)	0.07	0.07 (0.34)	0.22
4	Cumulative risk x lnRSA x PEP	−0.82 (0.84)	−0.98	0.99 (0.92)	1.07
Model 3: LnRSA response x PEP baseline					
1	Cumulative risk	1.26 (0.22)	5.72***	0.70 (0.25)	2.82**
2	lnRSA	−0.10 (0.25)	−0.40	0.24 (0.26)	0.93
	PEP	−0.00 (0.03)	−0.04	−0.04 (0.04)	−1.06
3	lnRSA x PEP	−0.02 (0.04)	−0.50	−0.07 (0.05)	−1.41
	Cumulative risk x lnRSA	0.19 (0.28)	0.67	−0.02 (0.29)	−0.06
	Cumulative risk x PEP	0.02 (0.03)	0.59	−0.02 (0.03)	−0.56
4	Cumulative risk x lnRSA x PEP	−0.11 (0.05)	−2.11*	−0.04 (0.06)	−0.74
Model 4: LnRSA response x PEP response					
1	Cumulative risk	1.12 (0.22)	5.09***	0.71 (0.23)	3.14**
2	lnRSA	−0.18 (0.23)	−0.78	0.16 (0.27)	0.62
	PEP	0.21 (0.27)	0.77	0.06 (0.23)	0.24
3	lnRSA x PEP	−0.14 (0.38)	−0.38	0.05 (0.36)	0.15
	Cumulative risk x lnRSA	0.08 (0.29)	0.29	−0.02 (0.33)	−0.07
	Cumulative risk x PEP	0.24 (0.32)	0.75	0.17 (0.32)	0.54
4	Cumulative risk x lnRSA x PEP	−0.91 (0.40)	−2.25*	−0.28 (0.42)	−0.67

*lnRSA* natural logarithm of respiratory sinus arrhythmia, *PEP* pre-ejection period

*†* < 0.10, *p < 0.05, **p < 0.01, ***p < 0.001

**Fig. 2:**
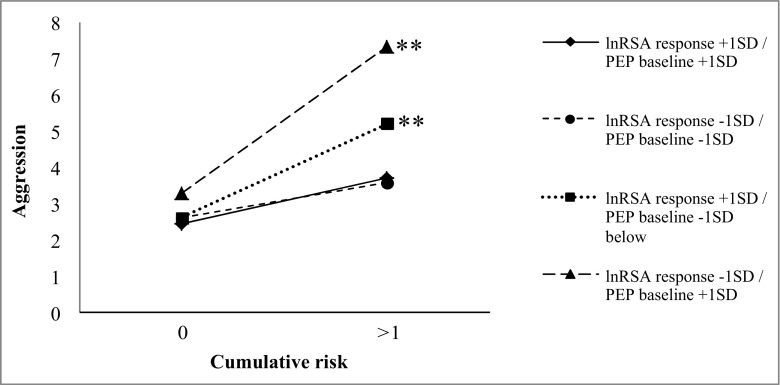
Three-way interaction between lnRSA response and PEP baseline, and cumulative risk, predicting physical aggression, **p < 0.01

**Fig. 3:**
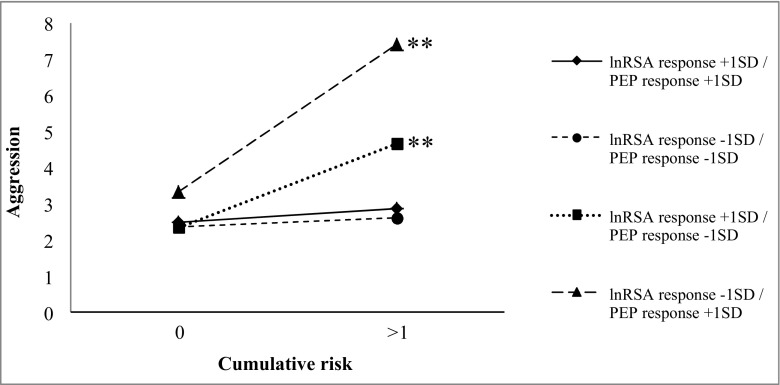
Three-way interaction between lnRSA and PEP response, and cumulative risk, predicting physical aggression, **p < 0.01

Significant three-way interactions were found between cumulative risk x lnRSA response x PEP baseline (*pr* = −0.21, *p* < 0.05) and cumulative risk x lnRSA response x PEP response (*pr* = −0.26, *p* < 0.05). Further examination of the three-way interaction between cumulative risk x lnRSA response x PEP baseline (see Fig. 2) revealed that higher cumulative risk predicted higher levels of physical aggression for infants exhibiting greater PNS suppression in response to stress (−1 SD; i.e., a decrease in lnRSA) combined with lower baseline SNS activity (+1 SD; high baseline PEP) (*β* = 1.08, *p* < 0.01), and for infants exhibiting greater PNS activation in response to stress (+1 SD; i.e., increase in lnRSA) combined with higher baseline SNS activity (−1 SD; high baseline PEP) (*β* = 0.69, *p* < 0.01). Conversely, for infants exhibiting greater PNS activation in response to stress (+1 SD) combined with lower baseline SNS activity (+1 SD) and greater PNS suppression in response to stress (−1 SD) in combination with higher baseline SNS activity (−1 SD), cumulative risk was not significantly related to physical aggression (respectively *β* = 0.33, *p* = 0.055, and *β* = 0.26, *p* = 0.143). Examination of the three-way interaction between cumulative risk x lnRSA response x PEP response (see Fig. 3) revealed that for infants exhibiting greater coinhibition (i.e., lnRSA response at −1 SD and PEP response at +1 SD) and coactivation (i.e., lnRSA response at +1 SD and PEP response at −1 SD) in response to challenge, higher cumulative risk predicted higher levels of physical aggression (respectively *β* = 1.09, *p* < 0.01, and *β* = 0.62, *p* < 0.01). Conversely, for infants exhibiting greater reciprocal PNS activation and SNS activation in response to challenge, cumulative risk was unrelated to physical aggression (respectively *β* = 0.10, *p* = 0.692, and *β* = 0.07, *p* = 0.722).

#### Non-physical Aggression/Oppositional Behavior

Results of the hierarchical regression analyses predicting non-physical aggression/oppositional behavior are shown in Table 2. The main effects for cumulative risk were in the same direction as in the hierarchical regression analyses predicting physical aggression, however the regression models were not significant after inclusion of the other predictors in steps 2–4.

## Discussion

The present study examined interactions between infant PNS and SNS functioning and prenatal adversity in predicting developmental outcome in toddlerhood. Our results align with theoretical models indicating that the complex associations between physiological functioning and behavior may be better understood as interactions with (early) adversity (Boyce and Ellis 2005; El-Sheikh and Erath 2011). Cumulative prenatal risk predicted physical aggression and non-physical aggression/oppositional behavior. However, significant two- and three-way interactions for physical aggression, but not for non-physical aggression/oppositional behavior, indicate that infant ANS functioning moderates this association. Specifically, the association between cumulative prenatal risk and physical aggression was present in children characterized by 1) low baseline PNS activity, and 2) nonreciprocal activity of the PNS and SNS, as evident by decreased activity (i.e., coinhibition) or increased activity (i.e., coactivation) at baseline and/or in response to emotional challenge. We found no moderating effects of ANS recovery measures.

### Moderation by Baseline PNS Activity

We found a significant two-way interaction between cumulative risk and baseline PNS activity predicting physical aggression. Consistent with previous work in school-aged children exposed to marital conflict and parental drinking problems (El-Sheikh 2001, 2005a), the infants in this study who exhibited lower baseline PNS activity and were exposed to higher cumulative prenatal risk showed higher levels of physical aggression. Our findings suggest that high baseline PNS activity may buffer against the effect of (early) adversity. However, others have argued that high baseline PNS activity may increase susceptibility to environmental influence, resulting in higher levels of problem behavior in the context of unsupportive environments (Conradt et al. 2013), and even lower (aggressive) problem behavior in more supportive environments (Conradt et al. 2013; Eisenberg et al. 2012). Although this seems inconsistent, it may be an effect of the type of risk factors with which the ANS interacts. The aforementioned studies (Conradt et al. 2013; Eisenberg et al. 2012) focused on the quality of the environment or caregiving context as adversity factor, whereas in the present study (and other previous studies in school-aged children; e.g., El-Sheikh 2005a; El-Sheikh et al. 2001) most infants were exposed to maternal psychiatric problems and substance (ab)use. Although we had a clear rationale for examining risk as a cumulative variable, different types of risk factors may impact or interact with the ANS in different ways. We found no evidence for this in our study in follow-up analyses with maternal psychiatric diagnosis or substance use as separate predictors in the regression models. However, Waters et al. (2016) found an interaction between ANS functioning and maternal chronic depression on externalizing behavior problems but not with overcrowded housing. Future studies should explore how different maternal and environmental risk factors interact with ANS functioning.

### Moderation by Interaction between PNS and SNS at Baseline and/or in Response to Stress

Our results extend prior research in school-aged children (El-Sheikh et al. 2009; Gordis et al. 2010) by demonstrating that coinhibition (i.e., PNS suppression accompanied with low baseline SNS activity or SNS suppression) and coactivation (i.e., PNS activation accompanied with high baseline SNS activity or SNS activation) at six months of life, predict physical aggression at 30 months, but only among infants exposed to elevated levels of prenatal adversity. Notably, our results indicate that coinhibition in context of adversity confers higher risk for physical aggression than coactivation in context of adversity. The group mean for infants exhibiting coinhibition was more than one standard deviation above the average physical aggression level reported in a large community sample of 24- and 36-month old children (Alink et al. 2006), whereas the group mean of infants exhibiting coactivation lay within one standard deviation of the mean reported by Alink et al. (2006).

The interaction effects of coinhibition and coactivation with prenatal adversity suggests that infants with a less adaptive ANS functioning at six months of age, may be more sensitive to negative effects of maternal depression and anxiety and substance (ab)use, and maternal psychological and caregiving distress due to limited social support, single parenthood, unemployment and financial problems. Nonreciprocal activation of the PNS and SNS may yield an ambivalent physiological response in which one branch of the ANS increases arousal whereas the other branch dampens arousal (Berntson et al. 1991). Coinhibition of the PNS and SNS in the present study was evident by PNS suppression in response to stress accompanied by low baseline SNS activity or SNS suppression in response to stress. According to the Polyvagal theory (Porges 2007; Porges and Furman 2011), PNS suppression equips the infant for action by withdrawing its inhibitory influence on the SNS. However, without joint activation of the SNS, there may be insufficient metabolic output to mobilize an effective behavioral self-regulatory response (El-Sheikh and Erath 2011). Conversely, in case of coactivation, where PNS activation in response to stress was accompanied by high baseline SNS activity or SNS activation in response to stress, the PNS fails to withdraw its brake on the SNS and instead stimulates the body into a calm state, reflecting poor regulation of high emotional and physiological arousal (El-Sheikh and Erath 2011). Over time, these patterns of coinhibition and coactivation may promote aggressive behavior, especially in environments that tend to elicit these behaviors more often.

It should be noted that the precise pattern of interactions between prenatal adversity and PNS and SNS measures of baseline and response differed from previous studies. Whereas El-Sheikh et al. (2009) and Gordis et al. (2010) reported significant interactions between baseline and response values of the PNS and response values of the SNS, our findings revealed the opposite, namely, significant interactions between PNS response and baseline and response values of the SNS. Noteworthy is that the interaction between PNS response and SNS baseline could not be tested in the study of Gordis et al. (2010) due to multicollinearity problems, so we do not know whether they might have found the same interaction effect as we did. However, given the scarcity of studies looking into PNS and SNS interactions, and the fact that the children in this study were much younger, this suggests that the pattern of interactions between baseline and response measures of the PNS and SNS needs further research.

Another point worth mentioning is that, although it was beyond our scope, and not possible due to statistical power limitations, it is important to also examine interactions among baseline and response levels within one system (e.g., PNS baseline x PNS response). In fact, previous studies in older samples have demonstrated that low baseline PNS activity in combination with PNS activation in response to stress predicted the highest level of delinquency (Hinnant et al. 2011). Including both between-system and within-system interactions in one model would potentially better reflect the complexity of the ANS in interaction with adversity in predicting developmental outcome.

### No Moderating Effects ANS Recovery Measures

Contrary to our expectations, ANS recovery measures did not moderate the impact of prenatal adversity on physical aggression. Although few studies to date have addressed ANS recovery from stress, there is some evidence that blunted PEP recovery increases the positive association between adversity between ages 0–15 years and antisocial behavior in boys at age 16 (Sijtsema et al. 2015). Further, a study in 4–7 year old children showed that impaired vagal recovery predicted poor emotion regulation to frustration (Santucci et al. 2008), underlying the importance of studying ANS recovery measures in future research.

### Strengths, Limitations and Future Directions

The present study has a number of strengths including the longitudinal design, the use of a heterogeneous sample, the measurement of both PNS and SNS activity and their interaction early in life, the examination of resting, reactivity and recovery measures, and the focus on both physical aggression and non-physical aggression/oppositional behavior. However, our findings should be interpreted in light of several limitations. First, we relied on maternal reports of physical aggression and non-physical aggression/oppositional behavior. Future studies should use multiple informants and include behavioral observations of early behavioral problems. Second, the physiological measures were only assessed at six months of age. Although previous studies (e.g., Alkon et al. 2011) have reported moderate stability of PEP and RSA during resting and challenging conditions from 6 to 60 months, lower stability was reported for reactivity measures and ANS reactivity profiles. This indicates that during the first few years of life, autonomic responses to stress are not yet fully developed, and therefore may be influenced by repeated exposure to environmental stressors. Future longitudinal investigations should examine the stability of coinhibition and coactivation across development and their association with early adversity and later aggressive behavior. Third, we measured adversity during the prenatal period and behavioral outcome at 30 months. Although we assumed that prenatal adversity would continue postnatally (Monk et al. 2012), different postnatal experiences may have influenced the development of physical aggression. Future studies should investigate both prenatal and postnatal risk and their interactions with ANS functioning on developmental outcome. Fourth, although the results of this study provide some important new insights, providing initial support for the role of PNS and SNS in increasing vulnerability to aggression in the context of adversity in early childhood, the findings should be considered exploratory, and require replication in future studies. Finally, it should be noted that it is unsure whether our findings generalize to higher risk samples, given that the level of cumulative risk in our sample was relatively low with 43% having one or more risk factors and only 21% having two or more risk factors. It is therefore likely that the study provides information regarding the impact of a normative distribution of adversity on development.

### Conclusion

In sum, our findings indicate that low baseline PNS activity and nonreciprocal activation of the PNS and SNS in infancy, with increased or decreased activity within both branches of the ANS at the same time, increase vulnerability for early physical aggression in the context of higher cumulative prenatal risk. Further, these effects were found to be specific for physical aggression, as opposed to a broader spectrum of difficult behavior (see also Baker et al. 2013; Burt 2012), possibly indicating a stronger biological basis for aggressive behavior, whereas non-physical aggression/oppositional behavior may be more environmentally determined. Notably, the interactions between the ANS and early adversity predicted physical aggression over and above the effects of observed behavioral distress and mother-reported temperament at six months. The results of this study add to our understanding of how physiological systems measured early in development increase susceptibility to early adversity and highlight the need to incorporate indices of both PNS and SNS functioning in order to elucidate its role in developmental processes leading to early aggressive behavior. The ANS is rapidly developing in the first year after birth (Porges and Furman 2011), thereby marking an important period of increased susceptibility to environmental influences, which, in turn, creates opportunities for interventions to prevent the development of aggressive behavior.

## Electronic supplementary material

ESM 1 (DOCX 17 kb)
