# Supplementary material for: Infant Parasympathetic and Sympathetic Activity during Baseline, Stress and Recovery: Interactions with Prenatal Adversity Predict Physical Aggression in Toddlerhood
Source: J Abnorm Child Psychol. 2017 Aug 7;46(4):755–68. doi: 10.1007/s10802-017-0337-y (PMC5899751; doi:10.1007/s10802-017-0337-y)
Supplement: Supplementary file 1 — (DOCX 17 kb) [file 10802_2017_337_MOESM1_ESM.docx]

**Online Resource 1**

Infant parasympathetic and sympathetic activity during baseline, stress and recovery: interactions with prenatal adversity predict physical aggression in toddlerhood

Table 1. *Descriptives for lnRSA and PEP baseline, stress response and recovery variables.*

|  | *N* | *M* | *SD* | Min. | Max. |
| --- | --- | --- | --- | --- | --- |
| LnRSA |  |  |  |  |  |
| Baseline | 118 | 3.39 | .44 | 2.34 | 4.50 |
| SFP Baseline | 116 | 3.40 | .37 | 2.39 | 4.23 |
| SFP Social stress | 115 | 3.22 | .42 | 1.97 | 4.10 |
| SFP Recovery | 115 | 3.27 | .46 | 1.97 | 4.27 |
| CS Baseline | 115 | 3.31 | .39 | 2.28 | 4.16 |
| CS Frustration | 110 | 3.30 | .53 | 1.92 | 4.44 |
| CS Recovery | 110 | 3.18 | .39 | 2.22 | 4.02 |
| PEP |  |  |  |  |  |
| Baseline | 113 | 64.23 | 6.11 | 45.67 | 76.00 |
| SFP Baseline | 105 | 62.91 | 6.26 | 44.13 | 76.89 |
| SFP Social stress | 109 | 61.71 | 7.11 | 43.02 | 76.89 |
| SFP Recovery | 99 | 61.72 | 7.36 | 40.99 | 79.01 |
| CS Baseline | 112 | 63.36 | 6.22 | 45.06 | 76.89 |
| CS Frustration | 100 | 61.79 | 6.79 | 45.00 | 76.00 |
| CS Recovery | 102 | 63.69 | 6.75 | 44.99 | 83.00 |

*Note:* lnRSA = natural logarithm of respiratory sinus arrhythmia, PEP = pre-ejection period, SFP = Still Face Paradigm, CS = Car seat.
